# LECOM (Lead Extraction COMplexity): A New Scoring System for Predicting a Difficult Procedure

**DOI:** 10.3390/jcm12247568

**Published:** 2023-12-08

**Authors:** Wojciech Jacheć, Dorota Nowosielecka, Bettina Ziaja, Anna Polewczyk, Andrzej Kutarski

**Affiliations:** 1 2nd Department of Cardiology, Faculty of Medical Sciences in Zabrze, Medical University of Silesia, 40-055 Katowice, Poland; 2 Department of Cardiology, The Pope John Paul II Province Hospital, 22-400 Zamość, Poland; 3 Department of Cardiac Surgery, The Pope John Paul II Province Hospital, 22-400 Zamość, Poland; 4 Department of Cardiology, Specialist Hospital, 41-800 Zabrze, Poland; 5 Department of Medicine and Health Sciences, The Jan Kochanowski University, 25-369 Kielce, Poland; 6 Department of Cardiac Surgery, Świętokrzyskie Center of Cardiology, 25-736 Kielce, Poland; 7 Department of Cardiology, Medical University, 20-059 Lublin, Poland

**Keywords:** complexity of transvenous lead extraction, difficult lead extraction, risk stratification

## Abstract

(1) Background: Transvenous lead extraction (TLE) can become far more complex when unanticipated difficulties arise. The aim was to develop a simple scoring system that allows for the prediction of the difficulty and complexity of this significant procedure. (2) Methods: Based on analysis of 3741 TLE procedures with and without complicating factors (extended fluoroscopy time, need for second-line instruments, and advanced techniques and instruments), a five-point Complex Indicator of Difficulty of (TLE) Procedure (CID-TLEP) scale was developed. Two or more points on the CID-TLEP scale indicate a higher level of procedure complexity. (3) Results: Patient age below 51 years at first CIED implantation, number of abandoned leads, number of previous procedures, passive fixation and multiple leads to be extracted, and a ratio of dwell time of oldest lead to patient age during TLE of >0.13 are significant predictors of higher levels of lead extraction complexity. The ROC analysis demonstrates that a point total (being the sum of the odds ratios of the above variables) of >9.697 indicates a 21.83% higher probability of complex TLE (sensitivity 74.08%, specificity 74.46%). Finally, a logistic function was calculated, and we constructed a simple equation for lead extraction complexity that can predict the probability of a difficult procedure. The risk of complex extraction (as a percentage) is calculated as [1/(1 + 55.34 · 0.754^X^)] · 100 (*p* < 0.001). (4) Conclusion: The LECOM score can effectively predict the risk of a difficult transvenous lead extraction procedure, and predicting the probability of a more complex procedure may help clinicians in planning lead removal and improving patient management.

## 1. Introduction

Transvenous lead extraction (TLE) plays a key role in solving problems related to intracardiac leads, such as infection, malfunction, upgrading, downgrading, abandonment, or venous occlusion. TLE is a highly effective procedure (the success rate is over 95%), with low major complication rates (1.6–2.5%) and a very low rate of procedure-related death (0–0.4%) [1,2,3,4]. The goal of the procedure is to remove all targeted leads safely (by a high-volume operator) and successfully (which depends on the organization of the procedure). The extraction should be performed from start to finish, even if there are unforeseen technical problems (e.g., no advancement of the dilator over the lead, fracture of the lead being extracted). Therefore, the final effectiveness of the procedure is determined by the operator’s experience and knowledge, as well as having all available auxiliary tools at hand (i.e., dedicated and non-dedicated removal tools [1,2,3]).

Pre-procedural assessment of the risk level associated with transvenous lead extraction procedures has been the subject of numerous reports [4,5,6,7,8,9,10,11,12,13]. First, the most important risk factors [3,4,5,6,7,8,9,10,11,12,13,14] were established, in order to identify the patients at highest risk of major complications related to TLE. Later, attempts were made to develop advanced scoring systems for more precise determinations of increased procedural complexity [10,13,14]. Finally, the ELECTRa Registry Outcome Score (EROS) was developed [13] and used for comparison, although the SAFeTY-TLE score for predicting the risk of major complications during TLE [14] seems to be most useful at present.

Much less attention has been paid to predicting the difficulty and complexity of the procedure, as these features have not been defined clearly enough. Several authors have proposed single variables as indicators of TLE complexity, such as fluoroscopy time [15,16,17] or the need to use advanced tools [18]. There are three approaches to the prediction of TLE complexity. Each approach is used to determine a individual indicator of procedure difficulty: the LED index (estimated fluoroscopy time) [19], the MB score (need for advanced tools) [20], and the Mazzone scale (need for advanced TLE techniques) [21]. The fact that we possess a database of 3741 TLEs prompted us to develop a model that helps to predict the level of lead extraction complexity and difficulty.

### Contributions of This Paper

In this retrospective observational study, we propose a simple approach for estimating procedural difficulty and transvenous Lead Extraction COMplexity (LECOM). Lead removal should be complete and free from complications and procedure-related deaths. Partial extraction or failure to extract leads is associated with a risk of infection and other secondary complications, generating additional costs. Predicting the probability that the procedure will be more difficult than usual may help clinicians make decisions about whether to refer patients to more experienced (i.e., high-volume) sites.

## 2. Aim of the Study

The aim of this study was to develop a validated risk score derived from a large database to predict TLE difficulty, measured in terms of prolonged lead extraction time/fluoroscopy time and the necessity of using second-line tools, advanced tools, and advanced techniques/approaches. In addition, we decided to construct an easy-to-use equation to estimate the risk of increased procedural complexity, which can help clinicians in planning TLE procedures.

## 3. Methods

### 3.1. Study Population

We reviewed a computerized database of transvenous lead extraction procedures performed between March 2006 and June 2022 by the same very experienced extractor at three high-volume centers. Patient characteristics, CIED and history of pacing, extracted lead information, TLE difficulty/complexity, efficacy, and outcomes were retrospectively analyzed. The study population consisted of 3741 patients (38.33% females) aged 5–97 years (average, 65.98 years).

### 3.2. Derivation and Validation Cohorts

Over the last 17 years, the organization of lead removal has evolved from procedures performed in the electrophysiology laboratory using intravenous analgesia/sedation [22] to procedures in the hybrid room under general anesthesia. For the last seven years, the core extraction team has consisted of the same highly experienced extractor (now frequently serving as a proctor), an experienced echocardiographer, and dedicated cardiac surgeon [15,18,22,23].

Odd-numbered procedures were retrieved from our prospectively collected database to form a derivation group, whereas even-numbered procedures were selected to form a validation group.

### 3.3. Lead Extraction Procedure

Indications for lead extraction, procedure effectiveness, and complications were defined according to recent recommendations (2009 and 2017 HRS consensus and 2011 and 2018 EHRA guidelines) [1,2].

### 3.4. Procedure Information

A standard step-wise approach was used in all patients, as previously described [14,15,16,17,18,19,20,21,22]. Lead dilatation was initiated using mechanical systems (i.e., polypropylene Byrd dilator sheaths; Cook^®^ Medical, Leechburg, PA, USA), primarily via the subclavian vein on the side of the implanted device. We used alternative vascular access and/or additional tools only if technical difficulties arose (e.g., Evolution [Cook^®^ Medical, Bloomington, IN, USA], TightRail [Spectranetix/Phillips, Colorado Springs, CO, USA], lassos, basket catheters). Laser and electrosurgical dissection sheaths were not used [14,22].

### 3.5. Procedure Difficulty: Complex Indicator of the Difficulty of the TLE Procedure (CID-TLEP)

First, we tried to define the concept of procedure complexity. Procedure difficulty (complexity) is expressed as procedure duration, in terms of an extraction time of all leads of >20.00 min (sheath-to-sheath time) or an average time of single lead extraction of >12.00 min (sheath-to-sheath time/number of extracted leads).

Unexpected difficulties (so-called “technical problems”) during TLE that increase procedure complexity but are not considered complications are another indicator of difficulty level. The most common technical problems in this context are lead-on-lead binding, fracture of the targeted lead, Byrd dilator collapse/torsion, and occlusion of the lead implant vein [14,15,16,17,18,19,20,21,22]. Other indicators of increased procedure complexity include no progress in lead dilatation, the occurrence of technical problems resulting in significantly prolonged procedure time, the need to use alternative venous access, and the need for more advanced tools and techniques, such as second-line tools (e.g., mechanical powered sheaths), advanced techniques (e.g., alternative approaches), and advanced tools (e.g., lassos); see Table 1.

### 3.6. Data Set and Statistical Methods

The 3741 extraction procedures performed between 2006 and June 2022 by the same very experienced extractor at three TLE centers were divided into two groups, depending on the patient’s consecutive number in the database; that is, a derivation cohort consisting of odd-numbered patients, and a validation cohort consisting of even-numbered patients.

Time of lead(s) extraction (sheath-to-sheath, or time of single lead extraction) (A or B) and significantly prolonged lead extraction time (C, D, E) were analyzed to determine the value (in points) of the Complex Indicator of the Difficulty of the TLE Procedure (CID-TLEP).

To identify the risk factors regarding TLE complexity, patients from the derivation cohort were sub-divided into two groups, according to the number of CID-TLEP points: CID-TLEP 0–1 and CID-TLEP 2–5 points. Continuous variables are presented as means and standard deviations, and non-parametric variables as medians and interquartile ranges (IQR). Categorical data are presented as counts and percentages. The significance of baseline inter-group differences was determined using the χ^2^ test, Student’s *t*-test, or unpaired Mann–Whitney U-test, as appropriate.

Receiver operating characteristic (ROC) curve analysis was performed to determine the optimal cut-off value for the ratio of the sum of oldest extracted lead dwell times to patient age during TLE and patient age at first CIED implantation. Depending on the cut-off values, the continuous data were then changed to dichotomous variables that took on the values 0 and 1.

Univariate and multivariate logistic regression analyses were conducted to assess the clinical and CIED-related factors that potentially increased the level of difficulty/complexity of lead removal. Uncorrelated variables with *p*-values < 0.05 under univariate analysis were entered into the multivariate logistic regression model. The regression results are reported as odds ratios with the corresponding 95% confidence intervals (CIs).

Points were assigned to each risk factor based on the odds ratio (OR). The total number of points served as a basis for developing a risk scoring system (LECOM) to predict the level of difficulty/complexity of the extraction procedure. Patients were divided into groups depending on the sum of points. In each sub-group, occurrence or no occurrence of difficulty/complexity (CID-TLEP points ≥ 2) was recorded. The data were then used to develop a risk curve. The logistic function was used to determine the relationship between the number of points and the probability of a difficult/complex TLE procedure. The validation of the LECOM score was based on the analysis of data obtained from 1870 consecutive patients undergoing lead extraction within the same period as patients in the derivation cohort.

ROC curve analysis was further performed to determine the number of LECOM points (for derivation and validation cohorts) above which the probability of technical complexity of the procedure significantly increased.

The Cochran–Mantel–Haenszel test was performed to check whether belonging to the derivation/validation group significantly affected the association between the probability of complex procedures and the number of points (testing only frequencies and not comparing logistic regression models that were constructed based on these frequencies). Additionally, in all patient groups, the predictive power of the developed LECOM score was compared with other scores estimating the probability of a complex extraction procedure, including EROS (increased risk of major complications and the need for cardiac surgery [13]), MB score (increased procedure complexity and the need for advanced tools to achieve TLE success [20]), SAFeTY-TLE score (increased risk of major complications during TLE [14]), LED index (difficult TLE, defined by fluoroscopy time [19]), and advanced TLE (Mazzone) score (the need for advanced TLE techniques [21]).

The *p* < 0.05 was consistent as the threshold of statistical significance. Statistical analysis was performed using the STATISTICA 13.4 software (TIBCO inc., Tulsa, OK, USA).

### 3.7. Approval of the Bioethics Committee

All patients gave their informed written consent to undergo TLE and to use anonymous data from their medical records, approved by the Bioethics Committee at the Regional Chamber of Physicians in Lublin no. 288/2018/KB/VII. The study was carried out in accordance with the ethical standards of the 1964 Declaration of Helsinki.

## 4. Results

Table 2 summarizes the baseline characteristics of patients in the derivation cohort (demographic data, indications for TLE, and CIED- and procedure-related information). Patients were divided into two sub-groups, according to the number of CID-TLEP points. The table shows that except for patient age at first CIED implantation and during lead extraction, patient-dependent factors such as female gender, NYHA class, diabetes, presence of renal failure, and infectious indications were not related to TLE difficulty. On the other hand, all CIED- and procedure-related data differed significantly between the groups. We also noted that the occurrence of major complications was higher in patients with CID-TLEP ≥ 2 points. In parallel, the rate of clinical and procedural success was significantly lower in these groups (Table 2).

Multivariate regression analysis in the derivation cohort demonstrated that the following factors were responsible for increased complexity of lead extraction (CID-TLEP ≥ 2): patient age below 51 years at first CIED implantation (OR = 1.362; *p* = 0.041), number of abandoned leads (OR = 1.558 for each; *p* = 0.013), number of previous CIED-related procedures (OR = 1.230 for each; *p* = 0.006), passive fixation leads to be extracted (OR = 1.649; *p* = 0.003), multiple leads to be extracted (OR = 1.921 for each; *p* < 0.001), and ratio of dwell time of oldest extracted lead to patient age during TLE of >0.13 (OR = 3.263; *p* < 0.001); see Table 3.

Based on the multivariate analysis, a scoring system was developed to predict the level of Lead Extraction COMplexity (LECOM); see Table 4.

**Figure 1:**
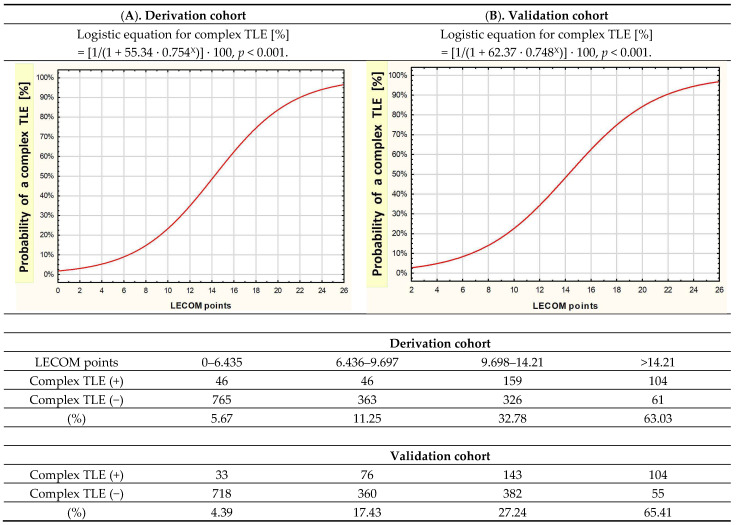
LECOM algorithm: relationship between the number of LECOM points and the probability of complex TLE, results obtained in the derivation cohort A (1871 patients) and evaluated in validation cohort B (1870 patients). Distribution of complex procedures in the derivation and validation cohorts depending on the number of LECOM points. Cochran–Mantel–Haenszel test *p* = 0.655. LECOM, lead extraction complexity; TLE, transvenous lead extraction.

The sum of LECOM points is correlated with the probability of a difficult/complex TLE procedure, where the relationship can be expressed as the logistic function in the following equation: probability of difficult/complex TLE (%) = 100/(1 + 644/(1.3213*x*)), where *x* is the number of points obtained (Figure 1).

The results of the Cochran–Mantel–Haenszel test (*p* = 0.655) indicated that the expected event rates in the validation group match the observed event rates in the derivation group. Therefore, for a fixed number of points, the probability of a complex procedure does not depend on belonging to the derivation or validation group (Figure 1).

**Figure 2:**
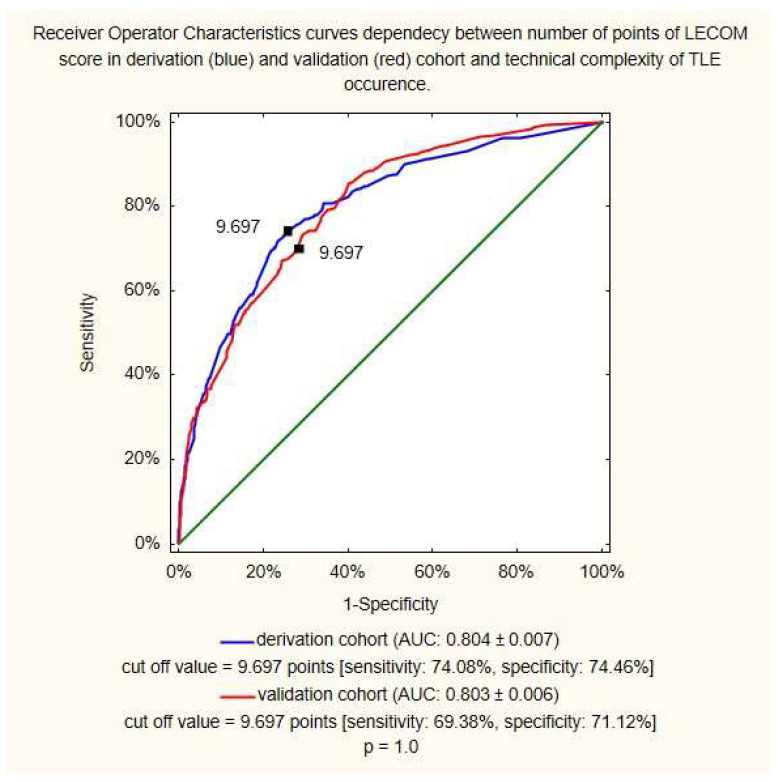
Relationship between receiver operator characteristic curves and LECOM score (points) in the derivation and validation cohorts. TLE, transvenous lead extraction; AUC, area under curve.

A LECOM score of 9.697 points was considered the cut-off value to indicate patients at a higher risk of undergoing a complex TLE procedure, with a sensitivity of 74.08% and specificity of 74.46% in the derivation cohort, and a sensitivity of 69.38% and specificity of 71.12% in the validation cohort (*p* = 1.0; Figure 2).

Next, we carried out a comparison of the new LECOM score with other scores for the prediction of complex or risky TLE procedures.

Other risk scores, similar to the new LECOM score, were significantly higher in the CID-TLEP 2–5 point group, when compared to the CID-TLEP 0–1 point group (Table 5).

Multivariable regression analysis demonstrated that the LECOM score (OR = 1.331, *p* < 0.001), LED score (OR = 1.080, *p* < 0.001), and Mazzone score (OR = 1.171, *p* = 0.046) performed best as predictors of difficult/complex extraction procedures (Table 5).

**Figure 3:**
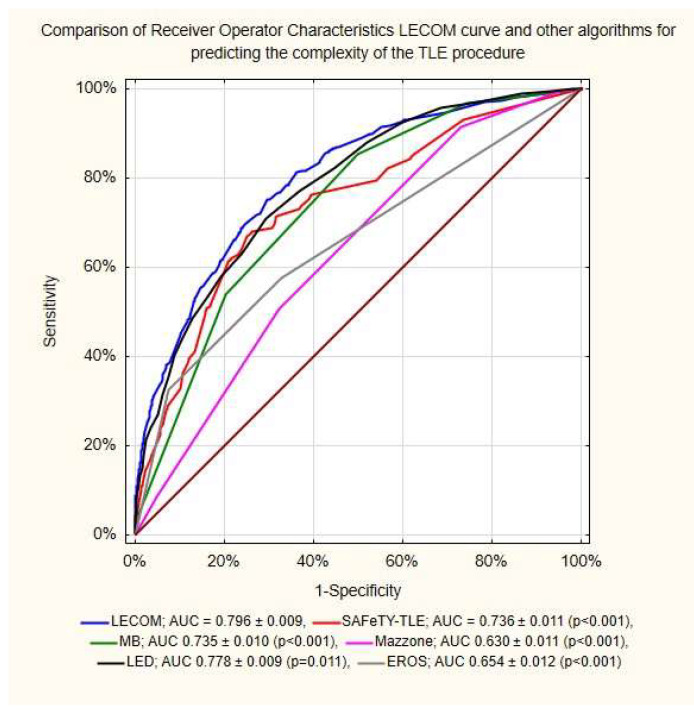
ROC analysis of LECOM and areas under the curves of other algorithms for predicting the complexity of lead extraction (n = 3741). LECOM, Lead Extraction COMplexity score; SAFeTY-TLE, SAFeTY-TLE score [14]; MB, MB score [20]; Mazzone, Mazzone score [21]; LED, LED index [19]; EROS, ELECTRa Registry Outcome Score [13].

The ROC analysis showed that compared to the other scores, LECOM was characterized by the significantly largest area under the curve as a predictor of difficulty/complexity of TLE (Figure 3).

## 5. Discussion

As the number of CIED implantations continues to increase, there is also a growing need for extraction procedures. New TLE facilities start as low-volume centers where extractors gradually gain experience [3,13]. Difficulties encountered during TLE may include no advancement of the dilator sheath, which requires switching to more effective tools, as well as fracture of the targeted lead, Byrd dilator collapse/fracture, lead-on-lead binding, need to change or use an alternative venous approach, loss of a broken lead fragment, and so on. All of these troublesome situations must be managed to achieve complete procedural success [19,20,21,22]. During basic training in lead extraction, it is not possible to learn how to cope with various problems as some of them occur relatively rarely. As a rule of thumb, the operator should complete the procedure in one stage, as performing the procedure in two stages significantly increases the risk of infection. Therefore, there is a need for a simple but reasonably effective equation to predict procedural difficulty. Patients whose lead extraction procedures are likely to be difficult should be referred to a high-volume center, allowing for care in the hands of the most experienced operators.

Lead tortuosity (higher values of Ottawa slack score) [15], the presence of lead-on-lead binding, adhesions between leads, veins, and heart structures [21,22,23], and passive fixation leads [20] are all predictors of an extraction procedure requiring advanced tools or prolonged fluoroscopy time [17,18].

There are several simple equations used in estimating the difficulty of a lead extraction procedure. There is the MB score, used to estimate the need for advanced tools (implant duration, number of extracted leads, passive fixation leads, ICD leads) [20]; the LED index, which predicts fluoroscopy time (number of leads to extract, dwell time of oldest lead to remove, presence of dual-coil ICD leads, and presence of vegetation along the lead) [19]; and the Mazzone scale, used to predict the need for advanced TLE techniques (patient age less than 71 years, age of the oldest removed lead > 37 months, removal of at least two leads, and removal of an ICD lead) [21]. So far, there has been no attempt to develop an equation that predicts both prolonged fluoroscopy time and necessity of using advanced tools and techniques (Figure 3).

We compared our new LECOM scale with the available risk scores for major complications or procedure complexity. Generally, there was an agreement between the results obtained with the SAFeTY-TLE score, EROS score, MB score, LED index, and the LECOM difficulty scale. The SAFeTY-TLE and MB scores showed higher compatibility with the LECOM scale than the EROS and Mazzone scores.

ICD leads are considered a risk factor for increased procedural complexity (MB score [20], LED score [19], and Advanced LE score [21]), due to possible externalization of the conductors hindering removal of the leads [23,24,25,26]. This insulation defect has been observed in St Jude Medical leads [26,27] and later Biotronik leads [23,24]. In countries where Riata leads are widely available, conductor externalization appears to be more frequent than elsewhere. In our country, Sprint leads (Quatro and Fidelis) were most often used at that time, whereas Riata leads were implanted only in a few patients. Consequently, there were fewer difficulties in extracting ICD leads. For this reason, ICD leads are not included in our calculation.

In-depth analysis of the available scores and our own material indicated that lead extraction complexity is predominantly determined by the following factors: age of the lead(s), abandoned lead burden, extraction of passive fixation leads, number of previous CIED-related procedures, and patient age at the time of device implantation and extraction. Other patient-dependent factors have a negligible effect (if any), contrary to popular opinion. Difficulties during lead extraction procedures (as well as procedural complications) are more common in younger people, those in better general health, and those without comorbidities. Unlike complications, gender has no impact on the level of TLE difficulty.

The new methods of pacing (His bundle pacing and left bundle branch area pacing) [27] create new challenges for lead extraction techniques. We extracted 30 conduction system pacing leads without major complications and without any particularly high degree of complexity of the procedure. However, this is too small a group to consider the location of the lead or the type of lead removed (Medtronic 3830) as a potential risk factor for the difficulty of the planned procedure.

The proposed LECOM scoring system seems to have practical value. In particular, patients with a score less than 2 may undergo lead extraction at a low-volume center, whereas those with higher scores should be referred to a high-volume site. The LECOM scoring system for determining the probability of a technically complex TLE (≥2 of Complex Indicator of the Difficulty of the TLE Procedure points) is available at www.usuwanieelektrod.pl/kalkulatory (accessed on 25 October 2023). 

## 6. Conclusions

The factors contributing to difficult lead extraction are comparable to the risk factors for major complications of the procedure. However, due to some differences, a special calculation approach is needed. Predicting the probability of a more complex procedure may help clinicians plan lead removal and improve patient management. The use of LECOM—a novel, user-friendly scoring system for predicting procedure complexity developed from a large cohort of patients undergoing transvenous lead extraction—can lead to improved procedural effectiveness.

### Study Limitations

Data were collected on a systematic and ongoing basis but analyzed retrospectively. Lead removal was performed using all types of mechanical systems, but not laser-powered sheaths. Thus, the results of this study are applicable to TLE procedures initiated using conventional first-line tools and techniques, when lead dilatation proves necessary.

## Figures and Tables

**Table 1:** The Complex Indicator of the Difficulty of the TLE Procedure (CID-TLEP).

	Determinants of TLE Difficulty	Points	Entire Group N = 3741	Derivation Cohort N = 1871	Validation Cohort N = 1870	Ch^2^ Test
			n (%)	n (%)	n (%)	*p*
A	Global sheath-to-sheath time for extraction of all leads > 20 min.	2	606 (16.20)	305 (16.30)	301 (16.20)	0.900
B	Average sheath-to-sheath time for extraction of a single lead > 12 min.	2	575 (15.37)	287 (15.34)	288 (15.40)	0.994
C	Necessity of using metal sheaths or Evolution/TightRail	1	281 (7.51)	147 (7.86)	134 (7.17)	0.460
D	Necessity of using alternative approach	1	127 (3.39)	69 (3.69)	58 (3.10)	0.368
E	Necessity of using lassos or basket catheters	1	155 (4.14)	80 (4.28)	75 (4.01)	0.745
CID-TLEP = (A or B) + C + D + E, CID-TLEP ≥ 2 = difficult extraction procedure						

TLE, transvenous lead extraction; N, number; CID-TLEP, Complex Indicator of the Difficulty of the TLE Procedure.

**Table 2:** Baseline patient characteristics in the derivation group, depending on occurrence or no occurrence of complex TLE.

	All N = 1871	CID-TLEP 0–1 Points N = 1516 (81.03%)	CID-TLEP 2–5 Points N = 355 (19.97%)	Chi ^2^/Mann–Whitney U-Test
	Mean ± SD n (%)	Mean ± SD Median [IQR] n (%)	Mean ± SD Median [IQR] n (%)	*p*
Patient age at first implantation [years]	57.85 ± 17.11 61.00 (19.00)	59.84 ± 15.70 62.00 (17.00)	49.34 ± 20.04 54.00 (29.00)	<0.001
Number of patients below 51 years at first CIED implantation	630 (33.67)	444 (29.29)	186 (52.39)	<0.001
Patient age during TLE [years]	66.15 ± 15.65 69.00 (17.00)	66.94 ± 14.80 70.00 (17.00)	62.77 ± 18.51 67.00 (27.00)	0.002
Female gender	730 (39.02)	606 (39.97)	124 (34.93)	0.079
NYHA class [I–IV]	1.83 ± 0.69 2 (1)	1.85 ± 0.69 2 (1)	1.78 ± 0.72 2 (1)	0.086
LVEF [%]	49.50 ± 15.35 54.00 (23.00)	48.85 ± 15.52 52.00 (25.00)	52.25 ± 14.34 56.00 (20.00)	<0.001
Atrial fibrillation	1074 57.40)	895 (59.04)	179 (50.42)	<0.001
Ischemic heart disease	822 (43.40)	707 (46.64)	115 (32.39)	<0.001
Diabetes mellitus type 2	393 (21.00)	329 (21.70)	64 (18.03)	0.126
Chronic kidney disease	419 (22.39)	336 (22.16)	83 (23.38)	0.597
Creatinine concentration [mg/dL]	1.22 ± 0.79 1.01 (0.42)	1.20 ± 0.71 1.00 (0.42)	1.28 ± 1.06 1.04 (0.44)	0.750
Charlson comorbidity index [points]	4.78 ± 3.70 4 (4)	4.92 ± 3.67 4 (4)	4.18 ± 3.76 3 (5)	<0.001
Infectious indications	595 (31.80)	481 (31.73)	114 (31.11)	0.889
Pacemakers (SSI, DDD, VDD, CRTP)	1348 (72.05)	1069 (70.51)	279 (78.59)	0.003
ICD (VR or DR)	407 (21.75)	358 (23.61)	49 (13.80)	<0.001
CRTD	135 (7.22)	101 (6.66)	34 (9.58)	0.072
Number of leads in the system	1.83 ± 0.62 2 (1)	1.81 ± 0.61 2 (1)	1.94 ± 0.66 2 (0)	0.004
Number of abandoned leads	0.15 ± 0.46 0 (0)	0.08 ± 0.32 0 (0)	0.42 ± 0.76 0 (1)	<0.001
Number of leads in the heart	1.96 ± 0.75 2 (0)	1.87 ± 0.66 2 (1)	2.35 ± 0.95 2 (1)	<0.001
Presence of abandoned leads	201 (10.74)	99 (6.35)	102 (28.73)	<0.001
Dwell time of oldest lead [years]	8.35 ± 6.25 7.00 (7.33)	7.19 ± 5.35 6.08 (6.67)	13.30 ± 7.34 11.25 (10.00)	<0.001
Number of previous CIED-related procedures	1.83 ± 1.03 2 (1)	1.67 ± 0.89 1 (1)	2.54 ± 1.28 2 (1)	<0.001
Extraction of unipolar leads	210 (11.22)	106 (6.99)	104 (29.30)	<0.001
Extraction of ICD leads	506 (27.04)	426 (28.10)	80 (22.54)	0.040
Extraction of passive fixation leads	1072 (57.30)	784 (51.72)	288 (81.13)	<0.001
Number of extracted leads	1.65 ± 0.73 2 (1)	1.54 ± 0.61 1 (1)	2.12 ± 0.97 2 (2)	<0.001
Dwell time of oldest extracted lead [years]	8.21 ± 6.17 7.00 (7.42)	7.04 ± 5.23) 6.00 (6.33)	13.23 ± 7.24 11.33 (10.25)	<0.001
Number of patients with dwell time of oldest extracted lead over 8.5 years	722 (38.59)	475 (31.33)	247 (69.58)	<0.001
Dwell time of oldest extracted lead/patient age at TLE (lead to age ratio) [years/years]	0.14 ± 0.13 0.102 (1.124)	0.12 ± 0.11 0.09 (0.10)	0.24 ± 0.17 0.20 (0.21)	<0.001
Number of patients with ratio of dwell time of oldest extracted lead to patient age during TLE > 0.13	701 (37.47)	449 (29.62)	252 (70.99)	<0.001
Technical problems (any) [yes/no]	368 (19.67)	149 (9.83)	219 (61.69)	<0.001
Number of technical problems	0.28 ± 0.68 0 (0)	0.11 ± 0.35 0 (0)	1.02 ± 1.10 1 (2)	<0.001
Procedure duration (skin-to-skin) [minutes]	59.70 ± 25.27 55.00 (18.00)	52.93 ± 14.11 54.00 (19.00)	88.60 ± 38.56 80.00 (35.00)	<0.001
Procedure duration (sheath-to-sheath) [minutes]	14.83 ± 22.61 9.00 (8.00)	7.49 ± 3.68 8.00 (5.00)	46.18 ± 37.76 35.00 (31.00)	<0.001
Average time of single lead extraction (sheath-to-sheath) [minutes]	8.68 ± 12.15 4.50 (5.00)	5.07 ± 2.24 4.00 (2.00)	24.11 ± 21.54 17.50 (15.00)	<0.001
Major complications	37 (1.98)	13 (0.86)	24 (6.76)	<0.001
Clinical success	1833 (97.97)	1502 (99.08)	331 (93.25)	<0.001
Complete procedural success	1787 (95.51)	1485 (97.96)	302 (85.07)	<0.001

TLE, transvenous lead extraction; N, number; CID-TLEP, Complex Indicator of the Difficulty of the TLE Procedure; CIED, cardiac implantable electronic device; NYHA, New York Heart Association functional class; LVEF, left ventricular ejection fraction; SSI, single chamber pacemaker; DDD, dual chamber pacemaker; VDD, dual chamber pacemaker (atrial sensing, ventricular sensing/pacing) with single ventricular lead; CRTP, cardiac resynchronization therapy pacemaker; ICD, cardiac implantable cardioverter defibrillator; VR, ICD single chamber; DR, ICD dual chamber; CRTD, cardiac implantable cardioverter defibrillator with resynchronization function.

**Table 3:** Derivation cohort: prognostic factors for procedure complexity; results of univariate and multivariate analysis.

	Univariate Regression			Multivariate Regression		
	OR	95% CI	*p*	OR	95% CI	*p*
Patient age at first implantation [1 year]	0.968	0.962–0.974	<0.001			
Patient age below 51 years at first CIED Implantation [1 year]	2.655	2.097–3.361	<0.001	1.362	1.012–1.832	0.041
Patient age during TLE [1 year]	0.984	0.978–0.991	<0.001			
Female gender	0.806	0.633–1.026	0.080			
NYHA class [1 class]	0.863	0.728–1.022	0.087			
LVEF [1% p.]	1.015	1.007–1.023	<0.001	1.007	0.997–1.019	0.162
Atrial fibrillation	1.065	0.814–1.392	0.648			
Ischemic heart disease	0.548	0.429–0.700	<0.001	0.843	0.609–1.166	0.302
Diabetes mellitus type 2	0.793	0.589–1.068	0.127			
Chronic kidney disease	1.077	0.818–1.418	0.597			
Creatinine concentration [1 mg/dL]	1.127	0.989–1.285	0.073			
Charlson comorbidity index [1 point]	0.944	0.913–0.967	0.001	1.023	0.979–1.070	0.304
Infectious indications for TLE	1.018	0.791–1.310	0.891			
Pacemakers (SSI, DDD, VDD, CRTP)	1.535	1.164–2.025	0.002	2.372	0.903–6.234	0.080
ICD (VR or DR)	0.518	0.375–0.716	<0.001			
CRTD	1.484	0.987–2.230	0.057			
Number of leads in the system [by 1]	1.403	1.165–1.690	<0.001	1.078	0.802–1.449	0.618
Number of abandoned leads [by 1]	3.454	2.748–4.341	<0.001	1.558	1.096–2.215	0.013
Number of leads in the heart [by 1]	2.269	1.938–2.658	<0.001			
Presence of abandoned leads	5.771	4.243–7.847	<0.001			
Dwell time of oldest lead [1 year]	1.154	1.132–1.177	<0.001			
Number of previous CIED-related procedures	2.063	1.845–2.307	<0.001	1.230	1.062–1.424	0.006
Extraction of unipolar leads [by 1]	3.172	2.456–4.089	<0.001	1.120	0.830–1.511	0.458
Extraction of ICD leads	0.744	0.566–0.978	0.034	1.230	0.888–1.705	0.213
Extraction of passive fixation leads	4.013	3.020–5.334	<0.001	1.649	1.188–2.290	0.003
Number of extracted leads	2.749	2.337–3.323	<0.001	1.921	1.475–2.500	<0.001
Dwell time of oldest extracted lead [1 year]	1.163	1.140–1.186	<0.001			
Ratio of dwell time of oldest extracted lead to patient age during TLE of >0.13	5.814	4.508–7.499	<0.001	3.263	2.324–4.582	<0.001

TLE, transvenous lead extraction; CIED, cardiac implantable electronic device; NYHA, New York Heart Association functional class; LVEF, left ventricular ejection fraction; SSI, single chamber pacemaker; DDD, dual chamber pacemaker; VDD, dual chamber pacemaker (atrial sensing, ventricle sensing/pacing) with single ventricular lead; CRTP, cardiac resynchronization therapy pacemaker; ICD, cardiac implantable cardioverter defibrillator; VR, ICD single chamber; DR, ICD dual chamber; CRTD, cardiac implantable cardioverter defibrillator with resynchronization function.

**Table 4:** Number of points predicting the level of lead extraction complexity (LECOM) based on multivariate analysis of TLE difficulty in the derivation cohort (1871 patients).

TLE Complexity	OR (LECOM Points)	95% CI	*p*
Patient age below 51 years at first CIED implantation	1.362	1.012–1.832	0.041
Number of abandoned leads (for each)	1.558	1.096–2.215	0.013
Number of previous CIED-related procedures (for each)	1.230	1.062–1.424	0.006
Passive fixation leads to be extracted	1.649	1.188–2.290	0.003
Number of leads to be extracted (for each)	1.921	1.475–2.500	<0.001
Ratio of dwell time of oldest extracted lead to patient age during TLE of >0.13	3.263	2.324–4.582	<0.001
**Probability of TLE complexity using the LECOM score**			
**LECOM points**	**RISK SCORE**		
<6.435	Low probability (<10.00%)		
6.436–9.697	Moderate probability (10.00–21.83%)		
9.698–14.21	High probability (21.84–50.00%)		
>14.21	Very high probability (>50.00%)		

LECOM, lead extraction complexity; TLE, transvenous lead extraction; CIED, cardiac implantable electronic devices.

**Table 5:** LECOM, MB, Mazzone, LED, EROS, and SAFeTY-TLE scores for prediction of difficult/complex extraction procedures.

Scores	All N = 3741	CID-TLEP 0–1 Points N = 3030	CID-TLEP 2–5 Points N = 711	Univariate Regression			Multivariate Regression		
	Mean ± SD Median [IQR]	Mean ± SD Median [IQR]	Mean ± SD Median [IQR]	OR	95%CI	*p*	OR	95%CI	*p*
MB [points]	2.58 ± 1.24 3 [2]	2.39 ± 1.23 2 [2]	3.40 ± 0.92 4 [1] *p* < 0.001	2.313	2.112 ÷ 2.532	<0.001	0.956	0.809 ÷ 1.130	0.276
Mazzone [points]	2.14 ± 0.92 2 [1]	2.06 ± 0.93 2 [2]	2.50 ± 0.79 3 [1] *p* < 0.001	1.728	1.570 ÷ 1.901	<0.001	1.171	1.003 ÷ 1.367	0.046
LED [points]	10.03 ± 6.39 9 [8]	8.74 ± 5.31 8 [6]	15.53 ± 7.59 14 [10] *p* < 0.001	1.170	1.154 ÷ 1.187	<0.001	1.080	1.050 ÷ 1.110	<0.001
EROS [points]	1.50 ± 0.71 1 [1]	1.41 ± 0.63 1 [1]	1.90 ± 0.86 2 [2] *p* < 0.001	2.425	2.176 ÷ 2.703	<0.001	1.062	0.916 ÷ 1.232	0.424
LECOM [points]	8.39 ± 4.17 7.78 [6.41]	7.49 ± 3.49 6.43 [4.90]	12.24 ± 4.62 11.91 [5.25] *p* < 0.001	1.331	1.299 ÷ 1.364	<0.001	1.232	1.184 ÷ 1.283	<0.001
SAFeTY-TLE [points]	5.85 ± 4.26 4.10 [6.10]	5.10 ± 3.78 4.10 [6.10]	9.07 ± 4.69 9.75 [7.45] *p* < 0.001	1.229	1.204 ÷ 1.254	<0.001	0.979	0.946 ÷ 1.013	0.227

CID-TLEP—Complex Indicatof of Difficulty of Transvenous Lead Extraction Procedure, LECOM, Lead Extraction COMplexity score; SAFeTY-TLE, SAFeTY-TLE score [14]; MB, MB score [20]; Mazzone, Mazzone score [21]; LED, LED index [19]; EROS, ELECTRa Registry Outcome Score [13].

## Data Availability

Readers can access the data supporting the conclusions of the study at www.usuwanieelektrod.pl (accessed on 25 October 2023).
